# Bridging surgical gaps in congenital heart disease: results from 50 remote telementored procedures using a new national digital platform in Brazil

**DOI:** 10.3389/fcvm.2025.1702387

**Published:** 2026-01-21

**Authors:** Rosangela Monteiro, Guilherme Rabello, Bianca Meneghini, Maria Carolina Guido, Luiz Fernando Caneo, Carla Tanamati, Maria Raquel Massoti, Leonardo Miana, Marcelo Marcos Morales, Ricardo Sgarbieri, Daniel Magalhães, Tamara Menezes Arruda, Camila Barbosa Rolim Cavalheira, Alfredo Ignácio Fiorelli, Vinicius José da Silva Nina, Marcelo Biscegli Jatene, Fabio Biscegli Jatene

**Affiliations:** 1 Department of Cardiovascular Surgery, Instituto do Coração, Hospital das Clinicas HCFMUSP, Faculdade de Medicina, Universidade de Sao Paulo, Sao Paulo, SP, Brazil; 2 Department of Innovation, Instituto do Coração, Hospital das Clinicas HCFMUSP, Faculdade de Medicina, Universidade de Sao Paulo, Sao Paulo, SP, Brazil; 3 Federal University of Rio de Janeiro, Rio de Janeiro, Brazil; 4 Santa Casa de Misericórdia de Passos, Minas Gerais, Brazil; 5 Hospital Metropolitano Dom José Maria Pires, João Pessoa, Brazil; 6 Hospital Universitário da Universidade Federal do Maranhão, São Luiz, Brazil

**Keywords:** pediatric cardiac surgery, telemedicine, cardiovascular surgical procedures, mentoring, distance counseling surgery

## Abstract

**Objective:**

Despite advances in cardiovascular surgery, access to specialized care remains limited in low-resource regions. Telemedicine, which expanded significantly during the COVID-19 pandemic, offers a strategy to mitigate these disparities. The National Teleconference Platform (TAC) was developed to provide real-time, multidisciplinary telementoring for pediatric congenital heart surgery in remote areas.

**Methods:**

This prospective, multicenter study assessed the feasibility, usability, and effectiveness of the TAC platform. The system integrated videoconferencing with Internet of Things (IoT) technologies, enabling synchronous audiovisual communication, real-time monitoring of surgical equipment, and interactive mentorship. Three Brazilian centers from the North, Northeast, and Southeast regions participated. Telementoring encompassed the entire surgical workflow, from anesthesia induction to postoperative debriefing, with data security compliant with national and international standards.

**Results:**

Between November 2022 and March 2025, 50 pediatric cardiac procedures were performed with complete telementoring in 93% of cases, and no technical failures occurred. Each operating room incorporated seven connected devices. User evaluations revealed high satisfaction: 76% of surgical teams reported increased confidence, 92% preserved autonomy throughout procedures, and all participants highlighted the substantial educational value of the platform.

**Conclusion:**

The TAC platform is feasible, effective, and well-accepted for providing multidisciplinary surgical guidance. It optimizes procedural performance, enhances surgical care, and demonstrates significant potential to expand access to specialized healthcare in resource-limited settings.

## Introduction

Access to high-complexity surgery remains unequal in low- and middle-income countries (LMICs), particularly in geographically isolated regions with limited medical infrastructure (1). In Brazil, pediatric cardiovascular surgery for congenital heart disease (CHD) exemplifies this disparity, as specialized centers and trained surgeons are concentrated in major urban areas, resulting in treatment delays, high referral costs, and increased mortality among vulnerable populations (2).

Infant mortality rate (IMR) is an indicator that reflects the living conditions of a population, particularly because children under 1 year of age are highly sensitive to socio-economic and environmental factors. Despite a decline in IMR over the decades in Brazil, the North and Northeast regions continue to face challenges, with the highest average IMRs (3).

Among the causes of infant mortality, congenital anomalies, particularly congenital cardiovascular malformations, account for about 40% of all congenital defects, thus significantly impacting infant mortality levels (4). In Brazil, the Ministry of Health reports that about 29 thousand children are born with CHD each year, with approximately 6% of these cases resulting in death before the first year of life (2).

Statistical data show that approximately 80% of CHD patients will require surgical intervention at some point in their lives, with only 20% experiencing spontaneous resolution of the condition. Around half of these cases will require surgery within the first year of life, resulting in an estimated demand of 11 thousand new pediatric cardiovascular surgeries annually in Brazil. The early treatment of CHD is crucial in preventing subsequent hospitalizations due to complications (5).

Telemedicine allows for the optimization of healthcare delivery, saving time and costs, while increasing access to medical care, enabling the delivery of specialized medical knowledge to remote locations and helping healthcare professionals to clarify questions regarding clinical procedures, health actions, and work processes (6, 7). Its rapid evolution during the COVID-19 pandemic further highlighted its value, and remote surgical guidance has emerged as an important tool in the transition of educational knowledge into the operating room (8).

However, an evident lack of research exists in the scientific literature regarding the development or application of a platform that offers specific functionalities for a functional and interactive interface between teams involved in surgery and highlighting the need for technological innovation (9, 10). The creation of a new platform designed for remote surgical mentoring stands as an innovative tool that can address the limitations associated with in-person surgical mentoring, while providing specialized support for surgical procedures in addition to reducing geographic spaces.

Therefore, the present study aims to develop and evaluate the effectiveness of the National Surgical Teleconference Platform (TAC) in providing real-time, remote surgical mentoring for CHD procedures. The study also sought to assess the feasibility, usability, and clinical impact of the TAC platform in geographically diverse healthcare settings, particularly in regions with limited access to specialized pediatric cardiovascular surgery.

## Methods

**Figure 1:**
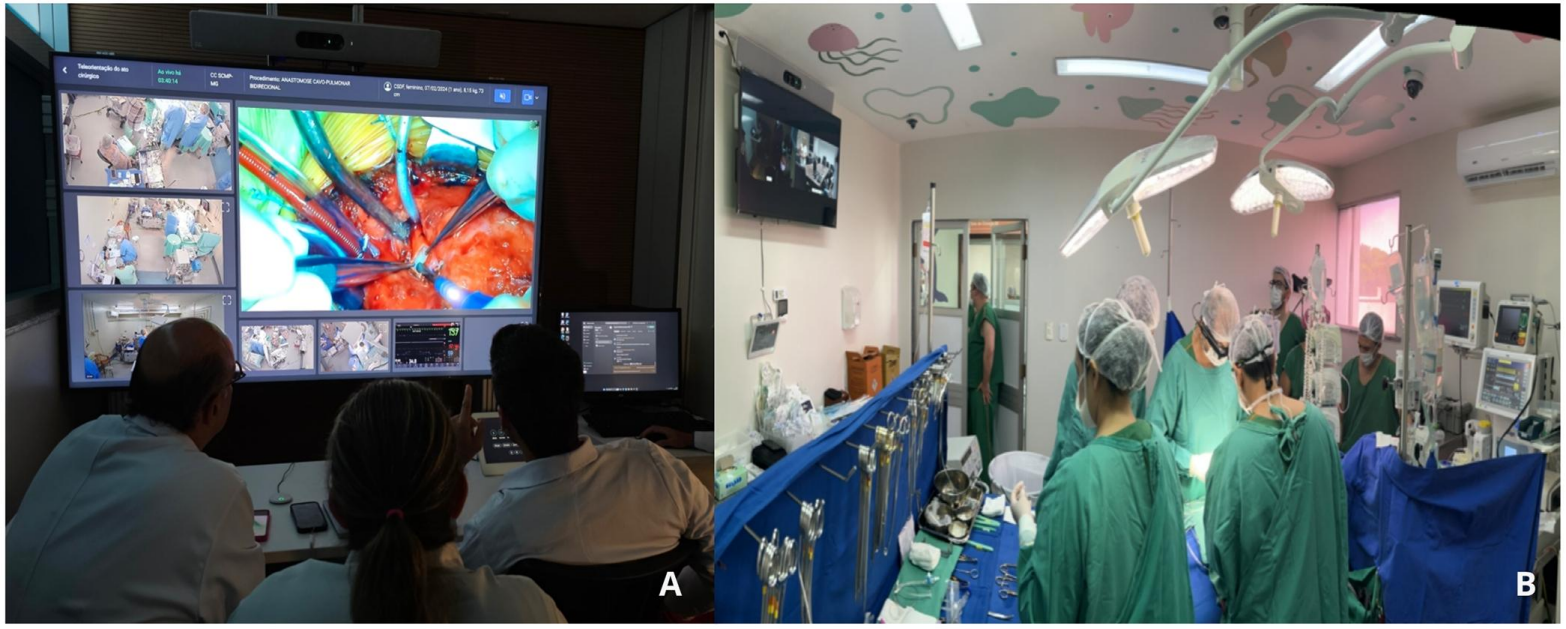
Teleconference platform (TAC) system. **(A)** Telementoring center located at our institution. **(B)** Telementored operating room in a hospital in the northern region of Brazil.

**Figure 2:**
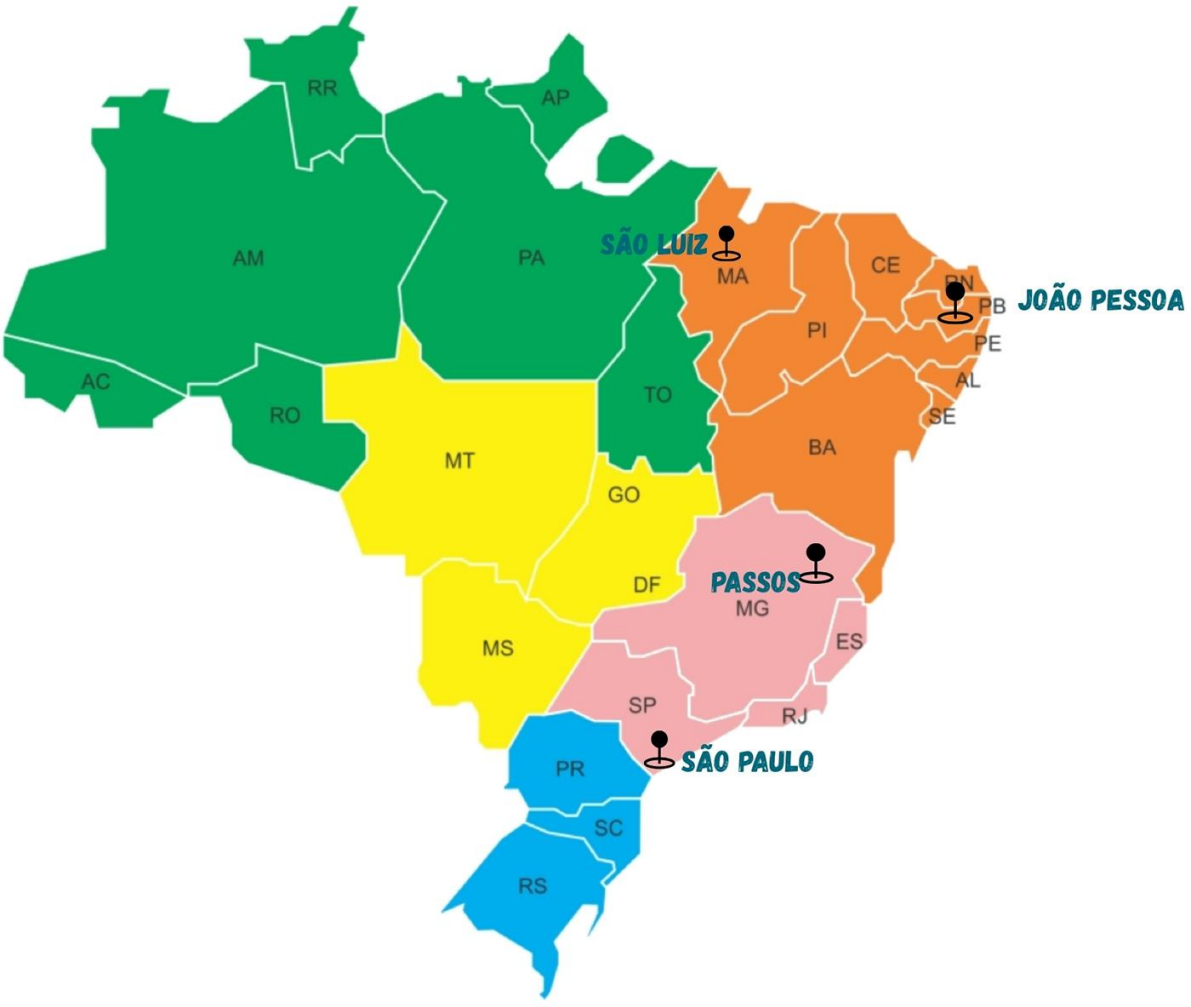
Teleconference platform (TAC) care network in Brazil. Brazilian regions currently supported by the TAC program.

This prospective, multicenter, feasibility study encompassed the development of the TAC platform and the establishment of two operational environments: the surgical telementoring room, based at our institution and named telementoring center, and the telementored room, in a hospital in the North region of Brazil (Figure 1). In the second phase of the project we expanded to another two locations; one in the Northeast region of the country and, the other, in the interior of the country (Figure 2). Cardiovascular procedures of different complexity were performed to treat CHD, with real-time, synchronous monitoring by a multidisciplinary team at the mentored center.

### Development of the national surgical teleconference platform

A platform was developed with functionalities that enabled a user-friendly, interactive interface. The platform framework was layered over Webex video conferencing platform (Cisco Systems, Inc.), which facilitates remote connectivity through audio and video calls. This allowed the integration of telementoring with technologies such as the IoT and artificial intelligence. Connections were established between the Webex platform and equipment used in routine surgery, such as multiparameter monitors, patient monitoring devices, and diagnostic tools. Additionally, images from cameras distributed in key areas of the telemonitored room were incorporated into the platform to monitor the surgical room comprehensively. All captured information was compiled into a unified dashboard, where the surgical procedure could be monitored in real-time, observing cameras and equipment signals simultaneously (Figure 1).

The platform was hosted on Cloud servers located at the Amazon AWS data center (Amazon Web Services, Inc.), which provided an individual, high-performance, non-shared environment with high availability. The infrastructure supporting the project included servers for Quality Assurance (QA), User Acceptance Testing (UAT), and production.

### Access to TAC, users, and inclusion of clinical data

Access to the TAC platform required registration of both the physician and institution to ensure data confidentiality. Only licensed medical professionals were eligible. Users were also registered on REDCap (11, 12) to input clinical data. The telementored hospital submitted surgical cases with reports, exams, images, and videos for expert review. Guidance was based on scientific evidence, national and international protocols, and local context. Supplementary materials, such as articles and surgical videos, were provided to support education and promote professional development of the monitored team.

At the end of each surgical procedure, a debriefing tool was employed, where the case was thoroughly discussed and analyzed. The physician from the monitored team conducted an evaluation of the telementoring process, considering its applicability and utility in clinical practice, as well as the resolution of the case in question.

### Setup of the telementoring center

A telementoring station was created to manage the platform and the process at the telementoring center. For teleconferencing, the Webex Room Kit Plus (Cisco Systems, Inc.) was used, connected to a high-performance computer equipped with image analysis and processing software, as well as broadband, high-performance internet. In addition, the telementoring station had access to the CAPES journal and scientific databases for consulting scientific articles, guidelines, and recommendations, enabling the telementoring team to contribute scientific evidence for their recommendations and proposed interventions.

### Creation and setup of the telementored room

The telementored hospital selected an operating room to set up the infrastructure. Fixed cameras were installed at strategic points pre-defined by the support team, as well as microphones for capturing ambient audio. Video codecs were used to capture data from the surgical equipment. Furthermore, a camera mounted on the surgeon's head was used to capture high-resolution images of the surgical field. All equipment was connected to a switch, enabling the transformation of data and generation of real-time images displayed on the dashboard. For teleconferencing, the Webex Room Kit Plus (Cisco Systems, Inc.) was also utilized.

### Telementored surgical procedure

Initially, to validate the TAC platform and integrate the surgical equipment with the platform, a pilot study was performed on with pediatric surgical procedures in a surgical room in the telementoring center. Subsequently, surgical procedures were performed at the telementored hospitals. The clinical cases were submitted and registered by the telementored hospital in the REDCap platform for evaluation by the telementoring center. The registration included pertinent clinical data, diagnostic findings, imaging exams, and medical reports. After the case was validated by the telementored team, surgeries were scheduled, and clinical cases were discussed in advance by the teams.

**Figure 3:**
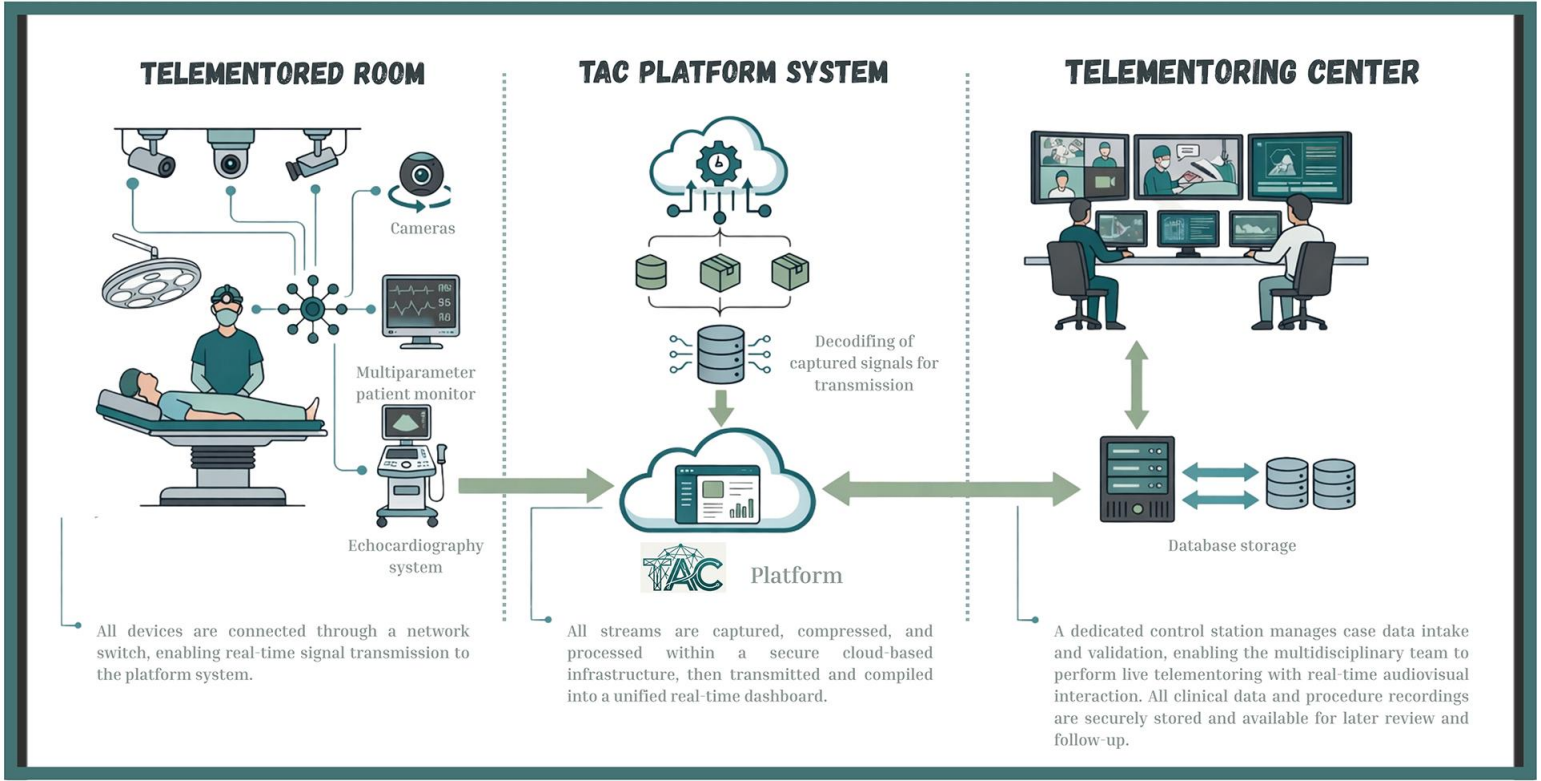
Telementored surgical procedure. Flowchart illustrating the workflow of a surgical telementoring system. The process begins at the telementoring center, where diagnostic data and medical reports are collected. Equipment signals and images from the telementored surgical room are then captured and transmitted via the teleconference platform to a referral hospital. The telementoring team can access this information in real time.

On the day of the surgeries, the telementored center team began telementoring even before the surgical procedure commenced, aiming to provide not just telementoring on the surgery itself, but on the entire surgical act. Multidisciplinary teams were able to interact synchronously and effectively throughout the procedures. After each procedure, an evaluation form was completed to document the monitoring interventions, and the experience of the multidisciplinary team was assessed. Post-operative clinical follow-up was conducted, and patient progress was accompanied with exams shared for ongoing evaluation (Figure 3).

### Structure and functionality of the TAC telemonitoring center

The use the platform enabled clear real-time communication between the teams, with no instability observed during the procedures. The TAC platform allowed for a functional and easy interactive interface between the telementored hospital and the telementoring center. The TAC platform successfully created, scheduled, and recorded the surgeries. After monitoring, all videos were archived for future review and surgical debriefing.

The platform supports two main telemonitoring features: live telementoring and recorded session review. Live sessions enable real-time interaction between mentoring and surgical teams via audiovisual communication, while guest users have view-only access. Recorded sessions offer on-demand playback with synchronized video, audio control, and timeline navigation. These features enhance procedural review, education, and knowledge sharing. The platform thus supports both synchronous and asynchronous learning, expanding access and improving training for surgical teams beyond the live operating environment.

### Storage and clinical data, ethical considerations

During the development of the platform, national and international guidelines concerning patient privacy and data security were strictly followed. An internal security policy was adopted, and confidentiality agreements were signed by all team members with access to the platform, ensuring full data protection. Access was granted through individual passwords with hierarchical levels, and communication occurred exclusively between the consulting physician and the multidisciplinary team at the telementoring center.

Patients or their legal representatives signed an informed consent form specifying their participation in the study and authorizing the sharing of their clinical information and diagnostic exams with the telementoring center team. For patients under 18 years of age, written informed consent was obtained from their parents or legal guardians. The study protocol was reviewed and approved by the Research Ethics Committee of HCFMUSP (Cappesq/SDC 5233/21/008). All procedures involving human participants complied with the ethical principles of the Declaration of Helsinki and its subsequent revisions, ensuring adherence to both national and international standards for research involving human subjects.

This project was designed as a prospective, multicenter, observational feasibility study. It did not involve randomization, control groups, or experimental interventions. Therefore, according to WHO/ICMJE definitions, it does not meet the criteria for a clinical trial and was not required to be registered in a primary clinical trial registry.

All clinical data related to the study were stored securely in the REDCap platform (Research Electronic Data Capture, Yale University). Data were anonymized upon completion of the study and descriptively analyzed by the telementoring center team.

### Patient selection

Patient of 12 years of age or less with a diagnosis of CHD and an indication for surgery were eligible. Exclusion criteria included the absence of clinical data and image exams.

The Risk Adjustment for Congenital Heart Surgery (RACHS-1) classification was calculated preoperatively and used to predict the mortality risk.

### Statistical analysis

For this descriptive feasibility study, continuous variables are presented as median [Interquartile Range—IQR] based on their distribution (normal/skewed), and categorical variables as counts and percentages.

Statistical analyses and graphical representations were performed using GraphPad Prism version 8.0 (GraphPad Software Inc., La Jolla, CA, USA).

## Results

### Use of the TAC platform in CHD procedures

Fifty surgical procedures were carried out between November 2022 and March 2025. Of these, 93% were fully telementored, with no platform downtime or network instability that could compromise the telemonitoring process. In three cases, there was a loss of internet connection due to mechanical failures in the local internet system, which prevented the completion of surgical follow-up; however, there was no harm to the patient.

Regarding infrastructure, seven pieces of equipment in each telementored room were connected to the TAC platform, including: 3 environmental cameras focused on the anesthetist, surgical scrub nurse and surgical team; one 360-degree camera providing a comprehensive view of the surgical room; one camera attached to the surgeon's head for viewing the surgical field; one multiparameter monitor; and one echocardiography device. This setup allowed, through the platform's drag-and-drop teleconference functionality, the entire surgical environment to be observed, enabling synchronous telemonitoring across all areas involved in the surgery.

The telementored procedure had an average duration of 5 h and 52 min, resulting in approximately 40 h of content per procedure. In total, more than 2,094 h of content were recorded. The average size of each video file generated was around 6.5 GB, leading to over 2.2 TB stored.

### Clinical data

A total of 50 patients underwent telementored surgical procedures. The majority of patients (39.78%) were referred from other cities, necessitating travel to the hospital, while 11 (22%) resided in the municipality where the hospital is located, underscoring the challenge of geographical access to specialized care.

The median age of the cohort was 3.5 years (IQR, 1.0–12.0) and 40% of patients were male. The prevalence of preoperative comorbidities was low, with reported occurrences of diabetes mellitus (2%), heart failure (12%), renal failure (2%) and leukemia (2%) (Table 1).

**Table 1:** Characteristics of the patients at baseline.

Characteristic	(*N* = 50)
Age (years)	3.5 [1.0–12.0]
Male sex, *n* (%)	20 (40%)
Clinical history	
Heart failure, *n* (%)	6 (12%)
Renal failure, *n* (%)	1 (2%)
Diabetes mellitus, *n* (%)	1 (2%)
Leukemia, *n* (%)	1 (2%)
Urea, [median (IQR)]	16.0 [13.0–17.0]
Creatinine, [median (IQR)]	0.4 [0.4–0.4]
Hemoglobin (Hb), [median (IQR)]	11.85 [11.3–12.3]
RACHS-1, *n* (%)	
1	13 (26%)
2	15 (30%)
3	13 (26%)
4	6 (12%)
5	1 (2%)
6	2 (4%)

Clinical data of patients undergoing telementored surgeries.

Surgical cases exhibited varying degrees of complexity, classified using RACHS-1 score (Table 1). Low-complexity procedures (RACHS-1 category 1) accounted for 12 cases (24%). Moderate-complexity procedures (RACHS-1 categories 2–3) constituted 30 cases (60%), encompassing interventions such as tetralogy of Fallot repair, valve repairs, valvuloplasties, and bidirectional cavopulmonary shunt (Glenn procedure). High-complexity procedures (RACHS-1 categories 4–6) represented 8 cases (16%) of the total cohort, including complex repairs for hypoplastic left heart syndrome, truncus arteriosus type I, and transposition of the great arteries utilizing the Damus-Kaye-Stansel technique combined with the Sano modification.

The most frequently performed interventions were repair of atrial septal defects and ventricular septal defects, each performed in 13 cases (26%). Repair of tetralogy of Fallot and bidirectional cavopulmonary anastomosis were performed in five patients each (10%), followed by atrioventricular septal defect repair in three cases (6%). Other complex procedures, performed less frequently (ranging from one to four cases), included total anomalous pulmonary venous connection repair, right ventricular outflow tract reconstruction with conduit implantation, cardiac valvuloplasty, truncus arteriosus repair, transposition of the great arteries using the Damus-Kaye-Stansel technique combined with the Sano procedure, and the Norwood-Sano procedure for hypoplastic left heart syndrome.

The surgical procedures had a mean duration of 4.5 h, with an average cardiopulmonary bypass time of 98 min and a mean myocardial anoxia time of 62 min.

The median time in the intensive care unit (ICU) was 4 days (IQR, 2.0–6.0), and the median length of stay was 4.5 days (IQR, 3.0–10.0). There was one intraoperative death in a high-complexity case undergoing repair using the Damus–Kaye–Stansel technique with the Sano modification. After weaning from CPB, the patient developed severe ventricular dysfunction that progressed to refractory cardiogenic shock despite appropriate resuscitative measures. Although the operation proceeded as planned, the patient's critical underlying condition contributed to the adverse outcome. Two patients experienced postoperative complications necessitating surgical reintervention.

### User experience assessment

After each surgical procedure, evaluation forms were filled out to document the telemonitoring interventions and assess the surgical team's experience, with the goal of determining the impact of real-time surgical mentoring on the surgeons.

Regarding the experience of the telementored hospital, 76% of the team reported complete confidence after the surgical briefing and reported improved communication with colleagues, greater effort during the procedure, and less impact on the patient. Additionally, 92% stated that the telementoring did not affect their autonomy or make them feel exposed during the process. All participants expressed satisfaction with the TAC experience, noting that it provided valuable learning.

## Discussion

This study presents the successful development and implementation of TAC, a telemedicine tool designed for remote, real-time surgical mentoring. The platform was applied to a population of pediatric patients undergoing congenital heart surgery in regions with limited access to specialized care. The findings demonstrate that TAC is technically feasible, clinically valuable, and capable of enhancing educational experiences for surgical teams in geographically distant locations.

The platform's innovation lies in its capacity to unify audiovisual communication and integrate surgical equipment signals into a single dashboard interface. This integration allowed for comprehensive real-time monitoring of the surgical environment, significantly improving the level of interactivity and clarity in communication between the telementoring center and the telementored hospital. Compared to traditional teleconference systems or passive video transmission, the TAC platform offers a more robust and immersive mentoring experience. Notably, the ability to review procedures asynchronously through recorded sessions supports ongoing education and quality improvement initiatives, which aligns with best practices in surgical education.

This study initially faced the challenge of defining the most appropriate method for connecting and transmitting data from the monitoring equipment linked to the patient within the surgical center. This challenge arises because such equipment is designed primarily for life support and is not necessarily optimized for internet connectivity or synchronous transmission. Additionally, the connectivity between the telementored hospital and the telementoring center needed to be stable enough to share real-time data, prioritizing which information would be shared and ensuring its availability so that the remote team could view parameters and videos as if physically present in the operating room, thus providing the best support possible.

In the context of CHD, a major contributor to infant mortality in Brazil, the impact of such a tool is considerable. Pediatric cardiovascular surgery requires high levels of specialization and institutional preparedness, both of which are unequally distributed across the country. By leveraging the TAC platform, institutions located in underserved regions were able to perform complex surgeries with real-time guidance from specialized centers. This not only enhanced local capacity but also contributed to reducing the need for patient transfers, which are often logistically and economically burdensome.

Currently, there is a deficit of specialized cardiac surgeons in Brazil, with most experts concentrated in metropolitan areas and major capital cities. This creates a significant regional disparity, often forcing patients and even specialists to travel in order to perform complex surgeries.

Clinical outcomes observed in this study reflect the potential of remote surgical mentoring to support safe and effective interventions. The predominance of high-complexity cases, the critical preoperative status of many patients, and the relatively low complication rate suggest that remote mentoring did not compromise, and may have contributed to, the safety and effectiveness of the procedures. While the study was not powered to evaluate mortality or long-term outcomes, the early indicators of safety and team confidence are promising.

Therefore, the TAC platform solution allowed significant advancements in training, offering several benefits: enabling real-time communication between local and remote teams, facilitating interaction during surgical procedures as if the remote team were in the operating room, and allowing recommendations for the local team. The consolidation of procedure data in the cloud ensured that a complete clinical record of the patient could be created and accessed by both teams for future interventions. The collected data and videos allowed for an analysis of the positive and negative aspects of the procedures, facilitating continuous improvement post-surgery. In the future, based on this experience, the solution could be expanded to other pediatric specialties and also has potential to support surgical care in adult patients with a broad range of cardiac diseases or other high-complexity conditions.

Importantly, user satisfaction data revealed that telementored teams valued the mentoring experience, citing increased confidence and enhanced communication as key benefits. Most participants did not feel that the process affected their autonomy or exposed them professionally. This finding is crucial, as resistance to external oversight can be a barrier to the adoption of tele-mentoring systems. The acceptance observed here indicates that the platform was perceived as a supportive educational tool rather than a supervisory constraint.

The results also emphasize the scalability of the TAC platform. The inclusion of multiple hospitals across different regions of Brazil, each with varying levels of infrastructure and human resource capacity, demonstrates the adaptability of the system. Furthermore, the use of cloud-based storage and modular architecture allowed for efficient data management and system scalability, features that are essential for national-level implementation.

This study has limitations. Although platform stability was high, reliance on internet access may hinder use in low-connectivity areas. Variability in participants’ soft skills—such as communication, teamwork, and adaptability—affected remote collaboration, requiring additional training. The initial implementation involved only three centers, limiting generalizability. While these sites were geographically diverse, the small scale underscores the need for broader deployment to validate the model across varied clinical and infrastructural settings. Expanded implementation will be essential to confirm its wider applicability.

## Conclusion

The successful implementation of a real-time telementoring platform for pediatric cardiac surgery in Brazil shows that low-cost, accessible technologies can overcome geographic and resource barriers in complex care. The platform supports decentralization, aligns with national digital health goals, and enhances access to specialized services. It also serves as an effective educational tool, strengthening local teams and enabling sustainable knowledge transfer. These results highlight its potential to transform pediatric cardiac care and be replicated in other high-complexity areas within public health systems.

## Data Availability

All data supporting the findings of this study are included within the article. Additional de-identified clinical and procedural datasets generated or analyzed during the study are available from the corresponding author upon reasonable request, subject to approval by the Institutional Ethics Committee. All data were securely stored in the REDCap platform and anonymized to ensure patient confidentiality.
